# Epidemiological and clinical based study on four passages of COVID-19 patients: intervention at asymptomatic period contributes to early recovery

**DOI:** 10.1186/s12879-020-05570-x

**Published:** 2020-11-17

**Authors:** Xiaohong Zhang, Hailian Wang, Youwei Wang, Yu Lei, Kaiju Xu, Jie Zhang, Ying Han, Jun Zeng, Shaoping Deng, Yi Wang

**Affiliations:** 1 Department of Emergency Critical Care Medicine, Sichuan Academy of Medical Sciences & Sichuan Provincial People’s Hospital, Chengdu, 610072 SC China; 2 Clinical Immunology Translational Medicine Key Laboratory of Sichuan Province, Sichuan Academy of Medical Sciences & Sichuan Provincial People’s Hospital, Chengdu, 610072 SC China; 3 Institute of Organ Transplantation, Sichuan Academy of Medical Sciences & Sichuan Provincial People’s Hospital, Chengdu, 610072 SC China; 4 Department of Respiratory Medicine, Chenfei Hospital, Chengdu, 610091 SC China; 5 Department of Critical Care Medicine, Ganzi Tibetan Autonomous Prefecture People’s Hospital, Kangding, 626000 SC China; 6 Translational Medical Center, Sichuan Academy of Medical Sciences & Sichuan Provincial People’s Hospital, School of Medicine, University of Electronics Science and Technology of China, Chengdu, 610072 SC China

**Keywords:** 2019 novel coronavirus (COVID-19), Transmission passage, Epidemiology, Clinical characteristics

## Abstract

**Background:**

With the worldwide spread of the 2019 novel coronavirus, scarce knowledge is available on the clinical features of more than two passages of patients. Further, in China, early intervention policy has been enacted since February. Whether early intervention contributes to swift recovery is still unknown. Hence, in this study, we focused on the patients from an isolated area, investigated the epidemiological and clinical characteristics of four serial passages of the virus.

**Methods:**

From January 25 to February 29, 2020, all patient data on the SARS-CoV-2 passages in this isolated area were traced, and the patients were grouped according to the passaging of SARS-CoV-2. Clinical characteristics of patients, including laboratory, radiology, treatment and outcomes, were collected and analyzed.

**Results:**

A total of 78 patients with four passages of virus transmission were included in this study. One patient transmitted SARS-CoV-2 to 8 patients (passage 2, P2), who next infected 23 patients (passage 3, P3), and then 46 patients (passage 4, P4). P2 received antiviral treatment when they had symptom, whereas P4 received antiviral treatment during their asymptomatic period. The incubation periods for P2, P3 and P4 patients were 7 days (IQR:2–12), 8 days (IQR:4–13) and 10 days (IQR:7–15), respectively. P2 patients showed lymphocytopenia (0.79 × 10^9^/L), decreased lymphocyte percentages (12.15%), increased white blood cell count (6.51 × 10^9^/L), increased total bilirubin levels (25% of P2 patients), increased C-reactive protein levels (100% of P2 patients) and abnormal liver function. By chest CT scans, all P2 patients (100%), 15 of P3 patients (65.22%) and 16 of P4 patients (34.78%) showed abnormality with typical feature of ground glass opacity. All of P2 patients (100%) received oxygen therapy, and in contrast, 19 of P4 patients (41.3%) received oxygen therapy. Further, significant decreased nucleic acid positive periods was found in P4 group (16 days, IQR: 10–23), compared with that of P2 group (22 days, IQR: 16–27). Moreover, the severity ratios were sharply decreased from 50% (P2 patients) to 4.35% (P4 patients), and the case fatality rate is zero.

**Conclusions:**

Judged from four passages of patients, early intervention contributes to the early recovery of COVID-19 patients.

**Supplementary Information:**

**Supplementary information** accompanies this paper at 10.1186/s12879-020-05570-x.

## Introduction

Since December 2019, the outbreak of 2019 novel coronavirus (COVID-19) has drawn considerable attention as a major public issue by World Health Organization (WHO) [1]. Up to March 3, 2020, the overall worldwide-confirmed patient number reaches to more than ninety thousand, and 73 countries have reported the confirmed cases [2, 3].

Accumulating evidence have revealed the clinical features of SARS-CoV-2 infected patients [4–6]. Until March 8, their investigation on the clinical features were limited to patients who were hospitalized with typical symptoms and these patients were carrying SARS-CoV-2 within two passages [7–12]. Scarce knowledge is available on the clinical features of patients with more than two passages of SARS-CoV-2. Further, since February, with the enactment of early intervention policy in China, the asymptomatic patients were hospitalized. Whether early intervention contributes to the early recovery was largely unknown to us.

Therefore, in the current study, we investigated on a special group of patients who lived in an isolated area. Due to the special geological features of this area, the epidemiological route of the SARS-CoV-2 transmission could be easily traced, and the patients were grouped according to the passages of SARS-CoV-2. In order to evaluate whether early intervention is beneficial to the patients, we compared the clinical characteristics of patients with or without treatment during their asymptomatic period, and found that early intervention may lead to the early recovery. To date, with around 40 million confirmed patients worldwide and with no effective antiviral agents targeting SARS-CoV-2, our study provide significant reference for the treatment of COVID-19.

## Methods

### Study design and data source

This study was approved by the institutional ethical committee of Sichuan Academy of Medical Science and Sichuan Provincial People’s Hospital. Due to the rapid spread of COVID-19, oral consents were obtained from patients by telephone, and the ethics committee approved the procedural for verbal consent. The first case of DaoFu county was reported on January 25, 2020 and the last case of this county was reported on February 27, 2020. The clinical outcomes were monitored up to February 29, 2020, and the final follow-up date was March 8, 2020. According to the clinical guideline of COVID-19 in China (version 7), all confirmed patients had symptoms and were positive for the virus with real-time reverse-transcriptase polymerase chain reaction (rRT-PCR) from pharyngeal swab specimens. The incubation period refers to the interval from the date of contact with confirmed patient to the date of disease onset, and the date of disease onset was defined as the day when the symptom was observed.

Basic information about age, gender, symptoms, comorbidities and epidemiology were collected. Meanwhile, the medical records, including clinical results (treatments and outcomes), laboratory data (the complete blood counts, chemical analysis, coagulation, liver and renal function, C-reactive protein, procalcitonin, lactate dehydrogenase, and creatinine kinase) and radiology results (chest computed tomographic scans) were also collected. The extracted electronic medical records were from Daofu County Hospital, Ganzi Hospital and Sichuan Provincial People’s Hospital. The research team from Institute of Organ Transplantation of Sichuan Academy of Medical Science & Sichuan Provincial People’s Hospital analyzed all medical records. In addition, the physician team from the Emergency Department of Sichuan Provincial People’s Hospital and Department of Respiratory Medicine, Chenfei Hospital, reviewed all the medical records. For the judgement of severe and critical ill patients, it was determined according to clinical guideline of Diagnosis and Treatment Protocol for NovelCoronavirus Pneumonia (Trial Version 7), released by National Health Commission and National Administration of Traditional Chinese Medicine of China. In brief, severe cases define to patients with respiratory distress (≧30 breaths/min), or oxygen saturation lower than 93% at rest, or arterial partial pressure of oxygen (PaO2)/ fraction of inspired oxygen (FiO2)≦300 mmHg. Cases with chest imaging that shows obvious lesion progression within 24–48 h > 50% were also defined as severe cases. Critical cases define to patients who had respiratory failure and requiring mechanical ventilation, or who had shock or other organ failure that requires ICU care [13].

### rRT-PCR assay

Pharyngeal swab specimens were collected, and rRT-PCR assay was performed at the Ganzi Autonomous Tibetan Preference Hospital and Center for Disease Prevention and Control, Ganzi Autonomous Tibetan Preference, Sichuan Province. In brief, after the collection of samples, total RNA was extracted using an RNA isolation kit (Tiangeng Biochemicals). Then the RNA suspension was used for rRT-PCR assay. The primers and probes sequences, reaction temperatures and protocols were in accordance with the China CDC guidelines for diagnose of SARS-CoV-2 at the WHO website (Supplement 1).

**Table 1:** Basic Characteristics of four passages patients

		Numbers (%)				
		Passage 1 (*n* = 1)	Passage 2 (*n* = 8)	Passage 3 (*n* = 23)	Passage 4 (*n* = 46)	*p* values
**Age**	Median	48.00	51.50	48.00	44.00	0.19
**Gender**						
Female		0	4 (50.00)	10 (43.48)	20 (43.47)	0.40
Male		1	4 (50.00)	13 (56.52)	26 (56.53)	
**Symptoms**						
Fever		1	7 (87.50)	16 (69.57)	26 (56.52)	0.19
Cough		1	8 (100.00)	18 (78.26)	38 (82.61)	0.36
Fatigue		1	8 (100.00)	23 (100.00)	44 (95.65)	0.50
Headache		0	1 (12.50)	2 (8.70)	1 (2.17)	0.32
Anorexia		0	1 (12.50)	4 (17.39)	12 (26.08)	0.56
Diarrhear		0	0 (0.00)	0 (0.00)	0 (0.00)	
Nasal congestion		0	0 (0.00)	1 (4.35)	0 (0.00)	0.30
Pharyngalgia		1	4 (50.00)	10 (43.48)	7 (15.22)	0.01
Lymphadenopathy		0	0 (0.00)	0 (0.00)	0 (0.00)	
Myalgia		1	1 (12.50)	1 (4.35)	7 (15.22)	0.41
Dyspnea		0	2 (25.00)	1 (4.35)	0 (0.00)	0.003
Vomit		0	0 (0.00)	1 (4.35)	1 (2.17)	0.77
Abdomin pain		0	1 (12.50)	1 (4.35)	0 (0.00)	0.10
**Comorbidities**						
Hypertension		0	4 (50.00)	6 (26.09)	9 (19.57)	0.18
Diabetes		0	4 (25.00)	3 (0.00)	1 (0.00)	< 0.001
Tuberculosis		0	3 (37.50)	1 (4.35)	3 (6.52)	0.01
Hepatitis B		0	0 (0.00)	1 (4.35)	2 (4.35)	0.83
Cardiovascular disease		0	0 (0.00)	1 (4.35)	0 (0.00)	0.30
COPD		0	1 (12.50)	0 (0.00)	0 (0.00)	0.01
Malignancy		0	0 (0.00)	0 (0.00)	0 (0.00)	
Chronic kidney Disease		0	0 (0.00)	0 (0.00)	0 (0.00)	
Chronic liver disease		0	0 (0.00)	0 (0.00)	0 (0.00)	
HIV		0	0 (0.00)	0 (0.00)	0 (0.00)	
**Medium incubation days**		9.00	7.00 (2–12)	8.00 (4–13)	10.00 (7–15)	0.005

*P* values indicate differences among Passage 2, Passage 3 and Passage 4 patients. *P* < 0.05 was considered statistically significant. The comparisons of the clinical characteristics, excluding age and incubation days, were performed by χ2 test. ^a^ The comparison of age and the comparison of incubation days among different groups was performed by one-way ANOVA with Bonferroni’s post-test

### Statistical analysis

Categorical variables were expressed as counts and percentages, and continuous variables were described as mean, median and interquartile ranges. Means for continuous variables were compared among three groups by one-way ANOVA with post hoc test, and they were compared between two groups by t tests. For the statistics of categorical variables, we performed χ2 test. When the data were limited, we performed Fisher exact test. All statistical analyses were performed by SPSS (Statistical Package for the Social Sciences) software (version 13.0). *P* <  0.05 was considered statistically significant.

## Results

**Fig. 1:**
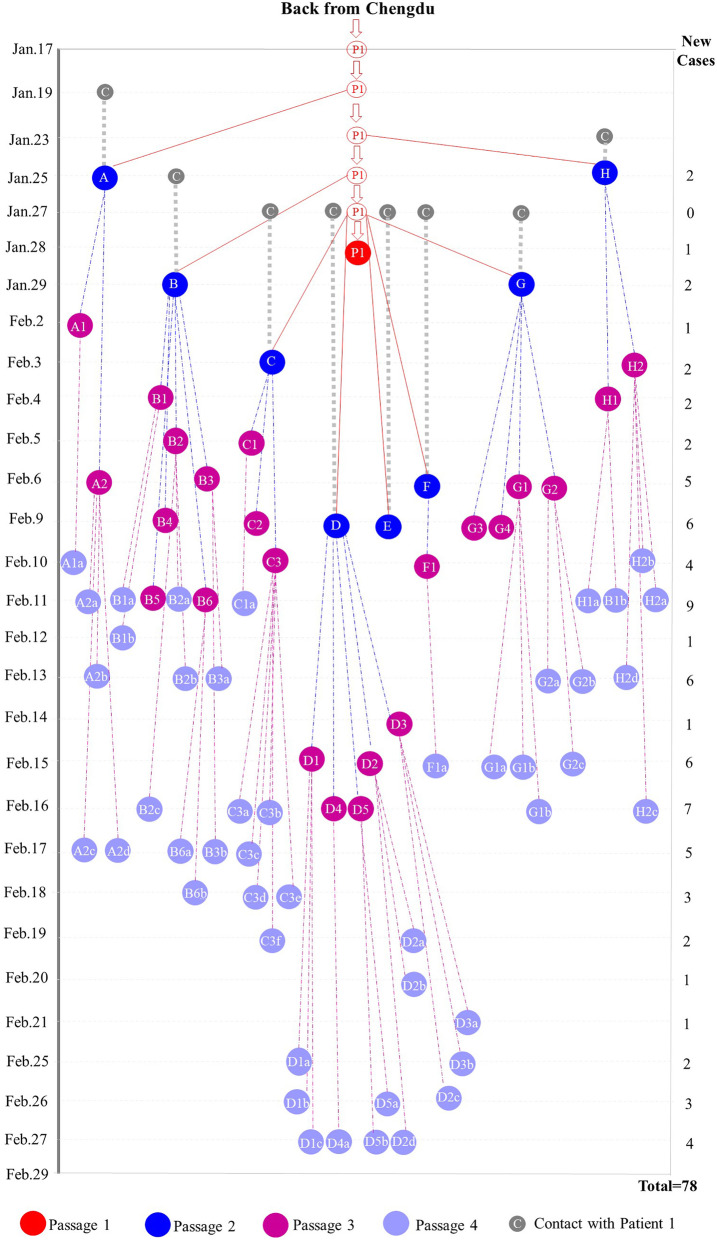
Transmission diagram for 78 confirmed patients infected with COVID-19. The red circle represents the asymptomatic carrier of COVID-19. The red ball represents the time that patient is confirmed positive by both symptoms and rRT-PCR assay. The grey balls with letter C means the contact time of eight patients (passage 2) with Patient 1. The dark blue balls represents eight patients of passage 2 (Patient A to H, respectively). The purple balls represents twenty-three patients of passage 3 (Patient A1 to H2, respectively). The light blue balls represents forty-six patients of passage 4 (Patient A1a to H2b, respectively). The grey dotted line represents the incubation time for passage 2 (Patient A to H). The red lines, blue dotted lines and purple dotted lines represent the transmission from passage 1 to passage 2, from passage 2 to passage 3 and from passage 3 to passage 4. Due to the comparatively large numbers of passage 3 and 4, the incubation time from passage 2 to passage 3 and from passage 3 to passage 4 is not included in this chart. The detailed information for the incubation time are listed on Table 1

Seventy-eight infected patients were found in a small county named DaoFu, which is an isolated area due to the geological features of Sichuan province, China. This county is far away from the downtown with around 60 thousand residents. Therefore, it is comparatively easy to track down all the patients with clear epidemiologic information. On January 17, 2020, Patient 1 (P1), an asymptomatic carrier of SARS-CoV-2, went to this county from downtown, where he had a close contact with a confirmed COVID-19 patient (P0). During his asymptomatic period, he infected 8 patients (passage 2, P2), who subsequently transmitted SARS-CoV-2 to 23 healthy people (passage 3, P3). Followed by the quarantine of the close contacts of P3, there were 46 SARS-CoV-2 carriers (passage 4, P4), who were confirmed positive by the nucleic acid tests. Taken together, with four passages of SARS-CoV-2 transmission, 77 patients were directly or indirectly infected by one single patient and the transmission route was shown in Fig. 1.

**Fig. 2:**
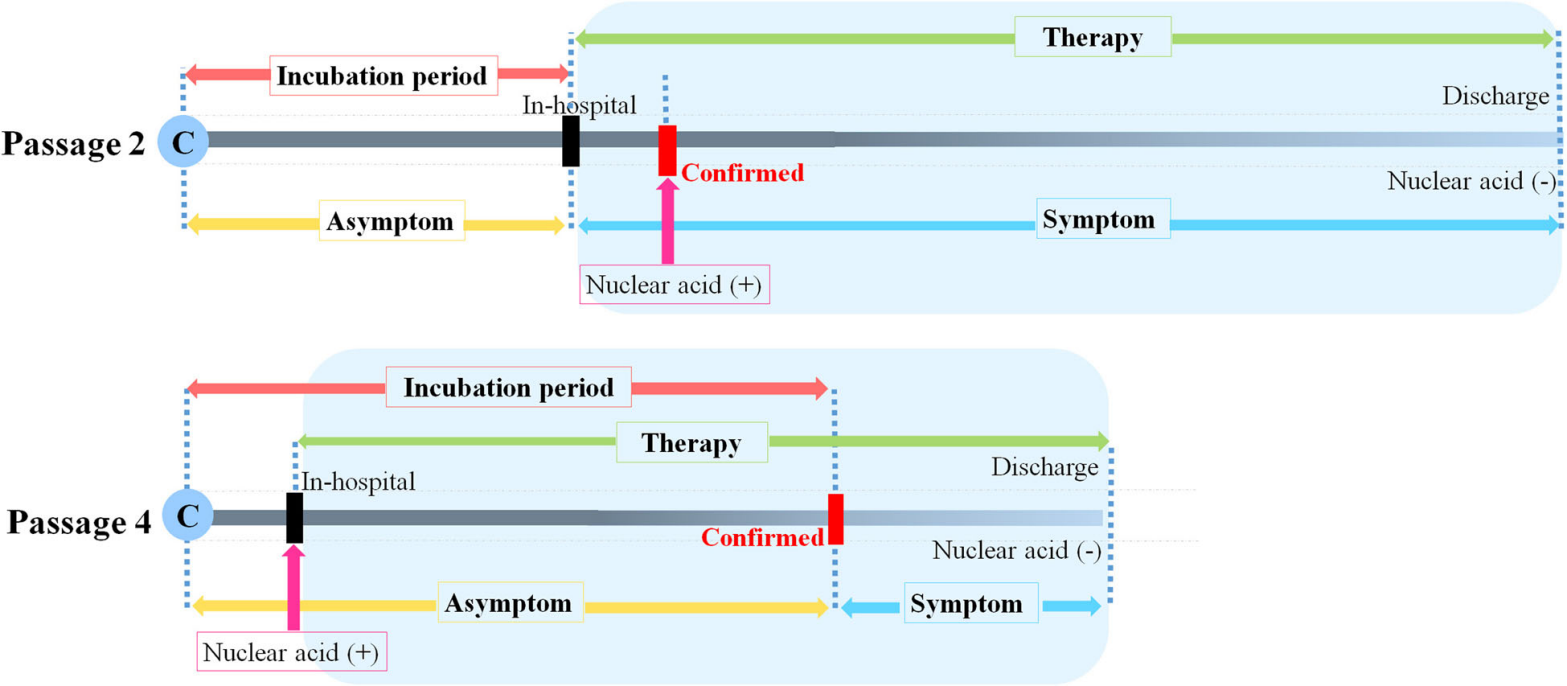
Schematic chart for time point from contact to discharge of P2 and P4 patients. C: contacting with confirmed patient; Nuclear acid (+): nucleic acid assay positive; Nuclear acid (−): nucleic acid assay negative; Confirmed time: the time confirmed to be COVID-10 patient

Since February, the Chinese government enacted the early intervention (including early diagnosis and early treatment) policy on all patients. Therefore, shown on Fig. 2, P2 patients received antiviral therapy when they had symptom and were hospitalized. In contrast, for the P4 patients, soon after their contact with confirmed patients, they received SARS-CoV-2 nucleic acid tests. Once positive for SARS-CoV-2, they were given antiviral therapy even though they were asymptomatic. Thereby, P2 patients and P4 patients were divided as the non-intervention group and the intervention group.

By comparing the baseline information of these 78 patients, we found that the median age for the 78 patients was 46.5 years, ranging from 2.7 to 77 years. The age distributions of four passages of patients, in-hospital patients and discharged patients were shown in Supplement 2 and 3 (Up to February 29). 43.6% of the patients were female, and no gender difference among the four passages of patients. Then we analyzed the symptoms and comorbidities of these patients. The major symptoms were fatigue (76 patients, 97.44%), cough (65 patients, 83.33%) and fever (50 patients, 64.10%). No patient has the symptom of diarrhear and lymphadenopathy. For the comorbidities, hypertension (*n* = 17), diabetes (*n* = 8) and tuberculosis (*n* = 7) were the most common diseases for them.

The incubation periods for P2, P3 and P4 patients were 7 days (range: 7–12 days), 8 days (range: 4–13 days) and 10 days (range: 6–15 days), respectively. Statistically, there is a significant difference for incubation periods (*P* <  0.05, by one-way ANOVA). Meanwhile, there is significant difference of incubation period between P2 and P4 by post hoc test (Dunnett’s multiple comparison test, *P* < 0.05). The overall incubation period was longer than previous reports. Then, we performed laboratory assay, and found that the P2 patients showed lymphocytopenia (0.79 × 10^9^/L, range: 0.40–1.10 × 10^9^/L, *P* < 0.05) and decreased lymphocyte percentages (12.15%, *P* < 0.05) (Table 2). Besides, the white blood cell counts of P2 patients increased to 6.51 × 10^9^/L (range: 3.40–7.90 × 10^9^/L, *P* < 0.05), which were significantly higher than those of P4 patients. Meanwhile, the neutrophil counts of P2 patients were also increased. The mean platelet counts of P2 patients was also lower than those of P4 patients. Further, index related with liver function as albumin and prealbumin were abnormal for P2 patients. We did not observe significant difference in ALT and AST levels among each group. Eight of P2 patients (100%), eleven of the P3 patients (47.83%) and thirteen of the P4 patients (28.26%) showed increased lactate dehydrogenase levels (*P* < 0.05 by χ2 test). Regarding the infection related index, two of the P2 patients (25%) showed increased total bilirubin (*P* < 0.05 by χ2 test), and eight of the P2 patients (100%) had increased C-reactive protein levels (*P* < 0.05 by χ2 test) compared with much lower percentages of 21.7 and 15.2% in P3 and P4 groups, respectively. One patient of P3 group, who are critical severe patient, had decreased creatine kinase (< 26 U/L). These laboratory data indicated that P2 patients might be in an exacerbated situation. Subsequently, we analyzed the severity of lung infection by chest CT results, found that all of P2 patients (100%) and fifteen of P3 patients (65.22%) showed abnormality with typical feature of ground glass opacity, which is statistically differently from that of P4 patients (34.8%, *P* < 0.05 by χ2 test).

**Table 2:** Laboratory, radiology findings and treatment of four passages patients

	Passage 1 (n = 1)	Passage 2 (n = 8)	Passage 3 (n = 23)	Passage 4 (n = 46)	*p* values
**Laboratory findings**					
**Blood count**					
White blood cell count, × 10^9^/L	4.60	6.51 (3.40–7.90)	5.34 (2.30–14.10)	5.21 (2.30–12.80)	0.04
Neutrophil count, × 10^9^/L	3.40	4.86 (1.60–11.40)	3.61 (1.10–12.20)	3.48 (1.00–7.70)	0.02
Neutrophil percentage	72.60	74.65 (45.80–91.60)	67.60 (28.40–87.10)	66.79 (34.60–84.50)	0.03
Lymphocyte count, ×10^9^/L	1.50	0.79 (0.40–1.10)	1.12 (0.50–3.10)	1.59 (0.60–2.90)	0.002
Lymphocyte percentage	22.00	12.14 (7.10–49.00)	20.97 (8.60–59.20)	30.51 (10.70–53.10)	0.02
Platelet count, ×10^9^/L	164.00	141.00 (65.00–214.00)	153.50 (57.00–272.00)	172.50 (63.00–311.00)	0.04
Prothrombin time, s	12.40	14.12 (11.50–16.00)	12.61 (11.40–14.10)	13.27 (11.30–16.90)	0.81
Red blood cell count(> 5.1 × 10^12^/L), No. (%)	0	2 (25.00)	2 (8.70)	1 (2.17)	0.04
**Blood chemistry** **Renal function**					
Blood urea nitrogen (< 2.8 mmol/L), No. (%)	0	2 (25.00)	5 (21.74)	4 (8.70)	0.23
Creatinine (> 123 μmol/L), No. (%)	0	0	1 (4.35)	0	0.30
Fibrinogen (> 4 g/L), No. (%)	2.64	3.08 (1.94–5.40)	2.75 (1.46–4.57)	2.34 (1.51–4.66)	0.26
Calcium, (< 2.1 mmol/L), No. (%)	0	2 (25.00)	2 (8.70)	2 (4.35)	0.13
Magnesium, (< 0.75 mmol/L), No. (%)	0	2 (25.00)	3 (13.04)	7 (15.22)	0.70
Sodium, (< 135 mmol/L), No. (%)	0	4 (50.00)	6 (26.09)	6 (13.04)	0.04
Chlorine,(> 108 mmol/L), No. (%)	0	0	1 (4.35)	2 (4.35)	0.83
**Liver function**					
Alanine aminotransferase (> 40 U/L), No. (%)	0	3 (37.50)	11 (47.83)	14 (30.43)	0.37
Aspartate aminotransferase, (> 40 U/L), No. (%)	0	3 (37.50)	9 (39.13)	9 (19.57)	0.18
Total bilirubin (> 28 μmol/L), No. (%)	0	2 (25.00)	1 (4.34)	1 (2.17)	0.03
Prealbumin levels (< 180 ng/L), No. (%)	0	5 (62.5)	10 (43.48)	9 (19.57)	0.02
Albumin (< 35 g/L), No. (%)	0	1 (12.50)	0 (0.00)	0 (0.00)	0.01
Lactate dehydrogenase, (> 245 U/L), No. (%)	1	8 (100)	11 (47.83)	13 (28.26)	< 0.001
**Infection related biomarker**					
Erythrocyte sedimentation rate (> 15 mm/h), No. (%)	0	6 (75.00)	7 (30.43)	11 (23.91)	0.02
C-reactive protein (> 10 ng/L), No. (%)	1	8 (100.00)	5 (21.74)	7 (15.22)	< 0.001
Procalcitonin (> 0.05 ng/mL), No. (%)	0	0 (0.00)	1 (4.34)	0 (0.00)	0.30
**Radiology findings**					
Ground glass opacity, No. (%)	1 (100.00)	8 (100.00)	15 (65.22)	16 (34.78)	< 0.001
**Treatment**					
Antiviral	1 (100.00)	8 (100.00)	23 (100.00)	46 (100.00)	
Antibiotics	1 (100.00)	8 (100.00)	6 (26.09)	9 (19.57)	0.001
Oxygen inhalation	1 (100.00)	8 (100.00)	16 (69.57)	19 (41.30)	0.002
**Severe and critical**					
Severed patient, No. (%)	0 (0.00)	4 (50.00)	2 (8.70)	2 (4.35)	< 0.001
Critical patient, No. (%)	0 (0.00)	0 (0.00)	1 (4.35)	0 (0.00)	0.30
Days in ICU, median, IQR	0	6.5 (3.5–9.5)	5 (3–8)	3 (2–4)	0.22

**Fig. 3:**
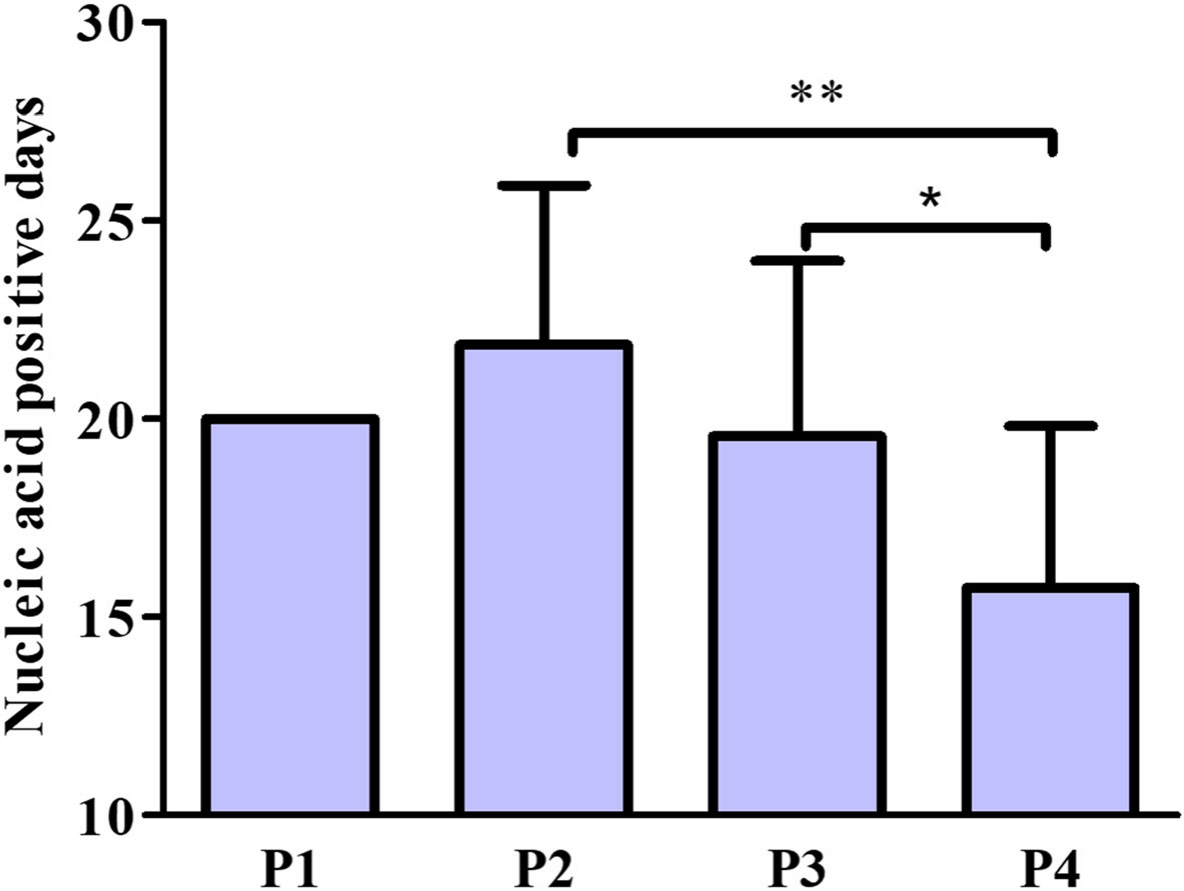
Comparisons of nucleic acid positive periods. *P* < 0.01 for comparison of nucleic acid from positive to negative among P2, P3 and P4 groups. **P < 0.01 for comparison of P2 and P4 groups, and *P < 0.05 for comparison of P3 and P4 groups. One-way ANOVA was performed with post hoc test

All the patients were given anti-viral drugs, and the majority of the patients were given antibiotics drugs and oxygen therapy. Eight of P2 patients (100%) received oxygen supplement, but only nineteen patients of P4 group (41.3%) received oxygen inhalation (*P* < 0.01 by χ2 test). Although the laboratory and radiology data cut-off date was February 29, 2020, in order to calculate the severe ratios, we performed the follow-up of the 40 in-hospital patients. Until March 8, 2020, there were only 7 in-hospital patients, and all of them were stable mild patients. So currently, the severe ratio of each passage was calculated on the formula, which was the number of severe patients of one passage divided by the number of total patients of the same passage. Interestingly, we found that four patients of the P2 group (50%) were severe patients, while two patients of P3 group (8.7%) and two patients of P4 group (4.35%) were severe patients. With the decreased percentage of severe patients in P4 group (*P* < 0.05 by χ2 test), it indicated that the treatment at early stage might lead to less severe patients. Besides, one patient of P3 was critical severe patient, who had been discharged from the hospital already. So, the overall severe ratio (10.26%) and critical ratio (1.28%) was significantly lower than the ratios of the reported cases in China (14% of severe and 5% of critical) [11]. In addition, we analyzed the ICU stay period for severe and critical ill patients, the median ICU stay period for P2 was longer than that of the P4. However, due to the limited number of severe and critical ill patients in each group, statistically, there was no significant difference in ICU stay. Up to now, the case fatality rate is zero. We also compared the nucleic acid positive periods of all discharged patients, and found that a significant decreased nucleic positive period was observed in P4 group (16 days, range: 10–23), compared with that of P2 group (22 days, range: 16–27) (Fig. 3).

## Discussion

To our knowledge, this is the first epidemiological and clinical evidence-based study on the four passages of SARS-CoV-2 in a clustered pattern. In particular, this study provides valuable clinical information of COVID-19 patients who received early intervention during their asymptomatic period. As the spread occurred in a rural area, the residents are sparsely inhabited far away from the downtown. Therefore, it was relatively easier to track down all the patients and their closely contacts. For that reason, we could identify each patient of this cluster and figure out the transmission passages.

Previous studies were mainly focused on cases reported at the initial spread of SARS-CoV-2 in China [7, 8, 12]. Meanwhile, they do not have clear transmission passages of their patients. Recently, a group of researchers did a comprehensive clinical analysis of 1099 patients, with 25% patient from Wuhan and 75% outside Wuhan [9], however, their data cut-off date was in January. According to a previous report, with the passages of virus and under the selective pressures, there might exist mutations of the virus [14]. However, with plenty of passaging of SARS-CoV-2 in patients from European countries and the United States, it seems that the infectivity and virulence were not decreased with the passages of the virus. As there is no effective anti-viral agents available, whether early intervention could alleviate the symptoms and lead to better outcome became a crucial question. Herein, in this article, our models of intervention group (P4) and non-intervention group (P2) at their asymptomatic period clearly indicated that early intervention might contribute to the early recovery.

For comparison of clinical data, we observed significant differences between P2 and P4 patients, probably due to the early intervention by antiviral drugs. Judged from our current data, earlier antiviral treatment may lead to the increased incubation period, decreased severity ratio and virus positive period. One possible explanation to the shorter incubation period of P2 patients is that those patients were unaware of the virus infection and did not receive any intervention until their body had to take measures against the virus (fever and cough). But for P4 patients, the virus replication is partially inhibited by the antiviral drugs especially when the virus load was at a comparatively lower level. Therefore, it might take longer time for the virus to reach the plateau phase from the exponential phase. According to the age distribution in supplement Table 2, P4 patients might be younger. Some studies revealed that elder patients were more prone to be critical ill, while some other studies have reported the potential for severe disease in the pediatric population [15–17]. It is possible that the better outcome of P4 patients might be attributed to the younger patients compared with those of P2 patients. In the current study, no critical ill patient was found in children or juvenile, and most patients were middle-aged adults. So there exist some possibilities that age distribution might not influence the final outcome. Further, to date, scarce knowledge is available for the infectivity and virulence of the virus with passages. Due to the discharge of these patients, we did not have the virus samples for sequencing. So, at current situation, we have no direct evidence on the virulence and infectivity of the virus. However, if the infectivity and the virulence of the virus were weaken with passages, the reported cases of Covid-19 would decrease worldwide. Taken the United States for an example, there was an outbreak of Covid-19 since this spring. To date, the daily reported new cases reached to more than 40,000. With plenty of passaging of the virus, the daily reported new cases would decrease if the virus became weaken. In fact, there is no tendency of decreasing in daily new cases. Therefore, the possibility for reduced infectivity is comparatively low. Besides, there were about 700 confirmed death daily in the United States. If the virus became less virulence, there would be less number of patients and fewer patients died of Covid-19. If the infectivity and virulence of the virus were stronger with passages, then judged from our data in Daofu county, early intervention was beneficial to the patients. Because, without the early intervention, the P4 patients would be in an exacerbated situation and had more severe/critical ill patients. If the infectivity and virulence of the virus maintained the same with passages, then early intervention was still beneficial to the patients. Because, P4 patients showed comparatively mild symptom, better laboratory results and good outcomes, comparing with those of P2 patients. The only reason that these patients had good outcomes is early intervention. Taken together, the good outcome of P4 patients might be attributed to the early intervention.

For the symptom, the differences between different groups were sore throat (*p* = 0.01) and dyspnea (*p* = 0.003). For laboratories findings of these two groups, P4 patients showed decreased WBC (*p* = 0.04) and neutrophil counts (*p* = 0.02), increased lymphocyte (*p* = 0.002) and platelet counts (*p* = 0.04). Compared with that of P2 patients, there were less P4 patients who had decreased prealbumin (*p* = 0.02), increased total bilirubin (*p* = 0.03), increased lactate dehydrogenase (*p* < 0.001), increased c-reactive protein (*p* < 0.001), increased erythrocyte sedimentation rate (*p* = 0.02). Therefore, according to the observations in this study, these symptoms and markers might be potential markers for severe patients. Another important clinical feature is that, due to the early diagnosis and early treatment, severe ratio of P4 patients (4.35%) was found to be significantly lower than that of total patients in China (14%) [11]. Due to seven of the P4 patients were still in hospital, we could not compare the discharge ratio. Hence, we compared the nucleic acid positive period, which was also the period for the virus to stay in the body. Interestingly, we found that virus stayed inside the body of P4 patients for a shorter time. One possible reason was that early treatment limited the virus replication, and this explanation further substantiated our findings on the incubation period.

There are still some limits in this study. Due to the far distance and other reasons, we did not keep enough samples of P1 and P2 patients to perform genetic sequencing. So up to now, we have no clue whether the virulence of SARS-CoV-2 in this group of patients were decreased. However, based on the clinical data, it seems obvious that the good outcome of the P4 patients could be attributed to the early intervention. The second limits is that due to the limit patient number we could hardly compare the antiviral drugs the patients received. There are three antiviral drugs they received, which are abidol, ribavirin and another Chinese medicine named Lianhua Qingwen granules. In this retrospective study, most of the patients received two of these drugs at that time.

## Conclusion

Based on our epidemiological and clinical studies of the four passages of SARS-CoV-2 infected patients, intervention at asymptomatic period contributes to the early recovery.

## Supplementary Information

**Additional file 1: Supplement 1**

**Additional file 2: Supplement 2**. Age distribution of four passages of patients, number (%)

**Additional file 3: Supplement 3**

## Data Availability

All data generated or analysed during this study are included in this published article.
