# Supplementary material for: Epidemiological and clinical based study on four passages of COVID-19 patients: intervention at asymptomatic period contributes to early recovery
Source: BMC Infect Dis. 2020 Nov 17;20:855. doi: 10.1186/s12879-020-05570-x (PMC7671187; doi:10.1186/s12879-020-05570-x)
Supplement: Supplementary file 1 — Additional file 1: Supplement 1 [file 12879_2020_5570_MOESM1_ESM.docx]

Supplement 1

For the rRT-PCR assay of COVID-19, both the open reading frame1ab (ORF1ab) and the nucleocapsid protein (N) were amplified and quantified. The primers and probes were listed below. For ORF1ab, the forward primer is 5’- CCCTGTGGGTTTTACACTTAA -3’, the reverse primer is 5’- ACGATTGTGCATCAGCTGA-3’, and the probe is 5'-FAM-CCGTCTGCGGTATGTGGAAAGGTTATGG-BHQ1-3'. For the nucleocapsid protein (N), the forward primer is 5’-GGGGAACTTCTCCTGCTAGAAT-3, the reverse primer is 5’- CAGACATTTTGCTCTCAAGCTG-3’, and the probe is 5'-FAM-TTGCTGCTGCTTGACAGATT-TAMRA-3'. For the real-time PCR assay, the reaction temperature, duration time and cycles are listed below. The reaction mixture was incubated at 50 °C for 15 minutes and 95 °C for 5 minutes. With 40 cycles of denaturation at 95°C for 15 seconds, the fluorescence signal was collected at 60 °C for 30 seconds (extending period).
