# Supplementary material for: Epidemiological and clinical based study on four passages of COVID-19 patients: intervention at asymptomatic period contributes to early recovery
Source: BMC Infect Dis. 2020 Nov 17;20:855. doi: 10.1186/s12879-020-05570-x (PMC7671187; doi:10.1186/s12879-020-05570-x)
Supplement: Supplementary file 2 — Additional file 2: Supplement 2. Age distribution of four passages of patients, number (%) [file 12879_2020_5570_MOESM2_ESM.docx]

| Supplement Table 2. Age distribution of four passages of patients, number (%) | | | | |
| --- | --- | --- | --- | --- |
| Age | Passage 1(n=1) | Passage 2(n=8) | Passage 3(n=23) | Passage 4(n=46) |
| 0-18 yrs | 0 | 0(0) | 2(8.7) | 8(17.4) |
| 19-49 yrs | 1 | 4(50.0） | 13(56.5) | 30(65.2) |
| 50-64 yrs | 0 | 2(25.0） | 6(26.1) | 5(10.9) |
| >65 yrs | 0 | 2(25.0） | 2(8.7) | 3(6.5) |
