# Supplementary figures and images for: Epidemiological and clinical based study on four passages of COVID-19 patients: intervention at asymptomatic period contributes to early recovery
Source: BMC Infect Dis. 2020 Nov 17;20:855. doi: 10.1186/s12879-020-05570-x (PMC7671187; doi:10.1186/s12879-020-05570-x)

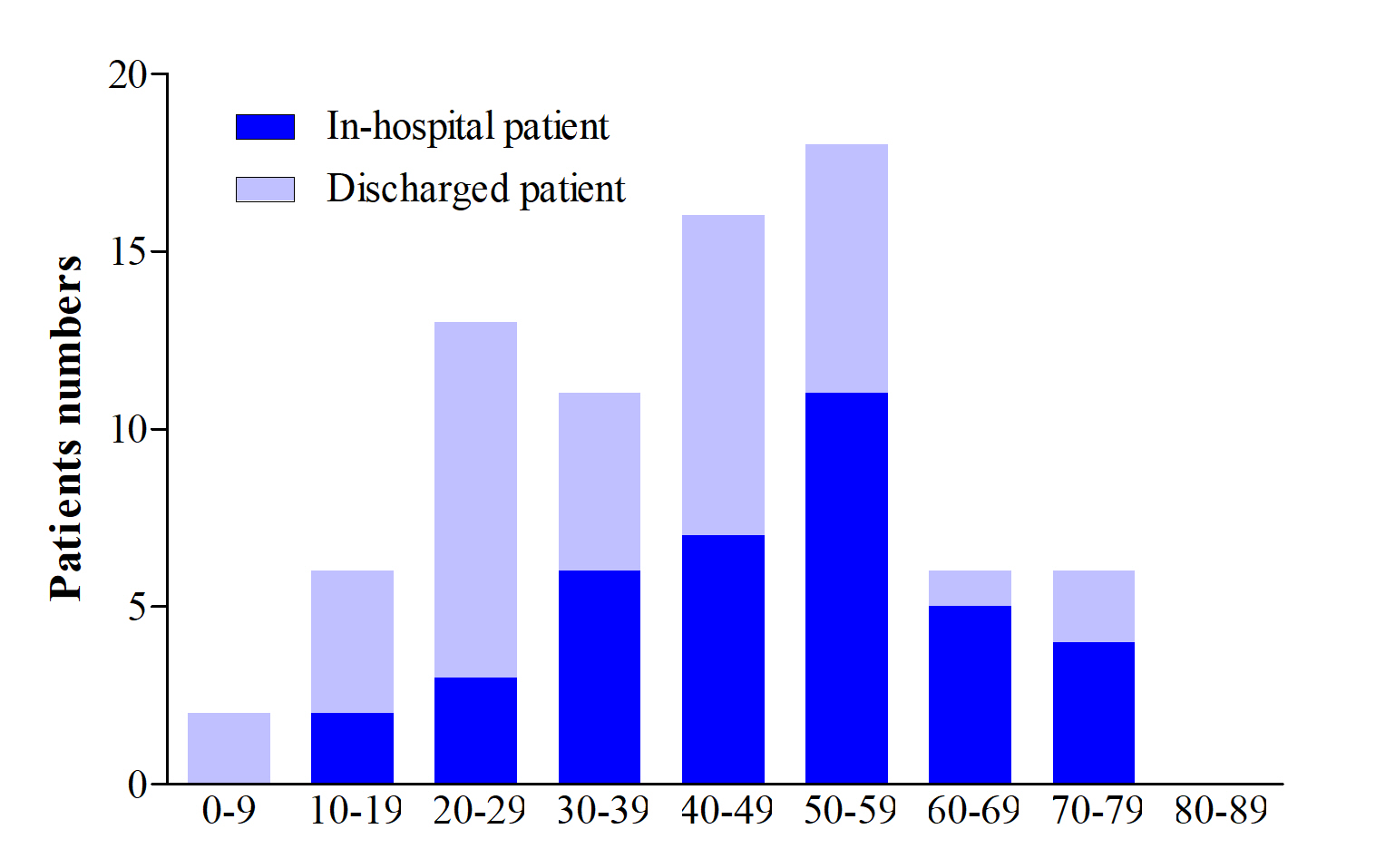

Supplement: Supplementary file 3 — Additional file 3: Supplement 3 [file 12879_2020_5570_MOESM3_ESM.jpg]
